# Adolescent reserve capacity, socioeconomic status and school achievement as predictors of mortality in Finland - a longitudinal study

**DOI:** 10.1186/s12889-017-4990-4

**Published:** 2017-12-28

**Authors:** Paulyn Jean Acacio-Claro, Leena Kristiina Koivusilta, Judith Rafaelita Borja, Arja Hannele Rimpelä

**Affiliations:** 1 Faculty of Social Sciences, Health Sciences, University of Tampere, Tampere, Finland; 2 Faculty of Social Sciences, Department of Social Research, University of Turku, Turku, Finland; 3 USC-Office of Population Studies Foundation, Inc. and Department of Nutrition and Dietetics, University of San Carlos, Cebu City, Philippines; 4 PERLA (Tampere Centre for Childhood, Youth and Family Research), University of Tampere, Tampere, Finland; 5 Pitkäniemi Hospital, Nokia / Department of Adolescent Psychiatry, Tampere University Hospital, Tampere, Finland

**Keywords:** Mortality, Socioeconomic status, Psychosocial resources, Reserve capacity, Life course epidemiology

## Abstract

**Background:**

Despite robust evidence on the inverse relationship between socioeconomic status (SES) and mortality, deviations from expected results have been observed likely due to school achievement and psychosocial resources, termed as “reserve capacity.” Since adolescence is a critical period in developing sound psychological and behavioural patterns and adolescent markers of SES were seldom used, we determine if family SES in adolescence predicts later mortality. We also study how reserve capacity (perceived health, health-promoting behaviour and social support) and school achievement modify this relationship and reduce the negative effects of low SES.

**Methods:**

A longitudinal study was designed by linking baseline data on 12 to 18 year-old Finns in 1985–95 (*N* = 41,833) from the Adolescent Health and Lifestyle Surveys with register data on mortality and SES from Statistics Finland. Average follow-up time was 18.4 years with a total of 770,161 person-years. Cox regression models, stratified by sex, were fitted to determine the effects of variables measured during adolescence: family SES, reserve capacity and school achievement on mortality risk.

**Results:**

All reserve capacity dimensions significantly predicted mortality in boys. Perceived health and social support predicted that in girls. Adolescents with the lowest school achievement were more than twice at risk of dying compared to those with better school performance. Low SES increased the risk of death in boys (Hazard ratios: 1.6, 95% CI 1.1–2.4) but not in girls. Reserve capacity and school achievement weakened the effects of low SES on boys’ risk of death.

**Conclusions:**

High reserve capacity and good school achievement in adolescence significantly reduce the risk of mortality. In boys, these also mitigate the negative effect of low SES on mortality. These findings underscore the roles of reserve capacity and school achievement during adolescence as likely causal or modifying factors in SES-health inequalities.

## Background

Research has extensively demonstrated the relationship between health and socioeconomic status (SES), often measured through income, education or occupation. Many studies have proven that low SES has adverse effects on health, acting cumulatively on morbidity and mortality [1–6]. A number of studies found high risks of premature death in both men and women with limited education, manual occupations and poor housing conditions [3, 5]. Also, regardless of adult socioeconomic status, poor socioeconomic conditions in early life were confirmed to be associated with mortality later in life [3, 5, 6]. Hence, SES has been proposed as a “fundamental cause” of health inequalities because it represents several resources like money, knowledge, prestige, power and beneficial social connections which can be used to improve health regardless of the disease mechanisms working at a given time [7]. Thus, even with improvements in medicine and other advances in health technologies, those without access to these resources lack the means to protect their health. This theory was empirically proven through a large study with a multi-country setting [8].

Despite the robust evidence on health disadvantages of low SES [1–6, 8], deviations have been observed [9–11]. Developmental studies have shown that early adverse exposures to poor environments could activate adaptive responses or mechanisms that provide long-term health advantages [12]. For example, early microbial exposure has been shown to boost immunity and increase resistance to diseases [13–15]. However, this field still warrants further testing and research.

Another perspective which likely explains said “epidemiological paradox,” initially described in distinct racial groups [16] is the psychosocial mechanism. Matthews and Gallo [9] proposed that individuals draw upon a bank of psychosocial resources called “reserve capacity” in response to acute and chronic stressors. Reserve capacity is a multidimensional concept which includes interpersonal resources such as social support and integration and intrapersonal characteristics such as self-efficacy, mastery or a sense of perceived control [9, 17–19]. We further extend the reserve capacity framework to include health behaviour since psychosocial resources underlie these factors and operate through them [20]. For instance, dental brushing behaviour and physical activity have been shown to improve with high self-efficacy [21–23]. Our study focuses on three dimensions: perceived health, health-promoting behaviour and social support.

Low reserve capacities trigger negative emotional and physiological responses and exacerbate the effect of low SES on cardiovascular morbidity and all-cause mortality via biological and behavioural intermediate pathways [9, 18, 19]. High reserve capacities decrease morbidity and mortality risks by regulating stress response, promoting positive emotions and facilitating adaptive coping which dampen pathogenic processes [11, 19]. For instance, some studies have attributed excess cardiovascular disease risk in low SES individuals to perceptions of weak job control [18]. On the other hand, accounting for self-efficacy reduced the risk of cardiovascular disease among those with low SES [17]. Low SES individuals with strong control beliefs and social connectedness had health outcomes similar to those of higher SES individuals [11, 19]. Conversely, increased risk to mortality were seen in those with reduced social resources [24].

There is a complex interplay of processes by which SES affects health throughout one’s lifetime. While family conditions determine early life SES and affect health outcomes in adulthood [3, 4], academic achievement in adolescence influences health, as well as current and future SES [25–27]. High achievement is associated with better health status and high SES [2, 25–27]. Decisions regarding school career leading to future adult education are affected by achievements in school [26, 27]. In addition, reserve capacity is shaped during adolescence [28]. Acknowledging these links, our study adopts a life-course approach [9, 29], where exposures during young adolescence are examined for their effects on the health trajectory, more specifically mortality.

Our aim is to study the relationship of family SES with mortality in adolescence and early adulthood. Moreover, we determine whether adolescent reserve capacity and school achievement contribute to mortality risk and modify the relationship between SES and mortality.

## Methods

### Study design

A longitudinal study was designed linking two data sources by means of unique national personal identification numbers. Baseline data were obtained from the Adolescent Health and Lifestyle Surveys (AHLS) of 1985, 1987, 1991, 1993 and 1995. Nationally representative samples of 12-, 14-, 16-, and 18-year-old Finns born on certain days in June, July and August were drawn each study year from the Population Register Centre. Overall response rate was 79% (*N* = 41,833), with 72% (*N* = 19,509) for boys and 86% (*N* = 22,324) for girls, respectively. A self-administered questionnaire was sent in February, followed by two re-inquiries to non-respondents. The variables used in our study were comparable across all survey rounds.

Follow-up data and information on family SES were respectively obtained from the Finnish Official Cause-of-Death Register and from the Register of Completed Education and Degrees, containing statistics on every resident in Finland. The follow-up started on 30 April, each survey year, and ended 31 December, 2009, or when the participant died. Average follow-up time was 18.4 years. It ranged from 1 to 25 years and had a total of 770,161 person-years. At the end of the follow-up, the participants were aged 27 to 43 years.

Statistics Finland performed the data linkage of the national registries and the AHLS data according to a contract specifying the rights and duties of both parties. The study protocol was approved by its Institutional Review Board and the Data Protection Ombudsman. The Joint Commission on Ethics of the University of Turku and the Turku University Hospital stated that no human rights were violated in the research protocol and approved it. Identification of the study participants was withheld from the investigators at all stages of the study. The first review boards at the universities were established in Finland in the 1980s. AHLS was reviewed by the Ethical Review Board of the University of Helsinki, Department of Public Health in 1986. Parental consent was not considered by the ethics review board at that time. In later surveys, the latest in 2017, the relevant review boards have waived the parental consent.

### Outcome and predictor variables

Table 1 shows the distribution of outcome and predictor variables. The outcome variable was death, defined by month and year. The predictor variables described family SES, reserve capacity and school achievement.

**Table 1:** Distribution of participants according to age at baseline, predictor variables and outcome status, Finland

Age at baseline and predictor variables in adolescence	Total population (*n* = 41,833)				Number of Deaths (*n* = 499)			
	Boys (*n* = 19,509)		Girls (*n* = 22,324)		Boys (*n* = 362)		Girls (*n* = 137)	
	No.	%	No.	%	No.	%	No.	%
Age at baseline (years)								
12	1976	10.1	1976	8.9	39	10.8	12	8.8
14	6011	30.8	6575	29.4	97	26.8	34	24.8
16	6285	32.2	7300	32.7	135	37.3	43	31.4
18	5237	26.9	6473	29.0	91	25.1	48	35.0
Family SES (parents’ education)								
Higher education	3261	16.7	3573	16.0	35	9.7	19	13.9
Secondary education	11,818	60.6	13,530	60.6	211	58.3	77	56.2
Basic or lower	4425	22.7	5210	23.3	116	32.0	41	29.9
No data	5	0.0	11	0.1	0	0.0	0	0.0
Perceived health								
Chronic disease								
No	17,791	91.2	20,134	90.2	312	86.2	119	86.9
Yes	1718	8.8	2190	9.8	50	13.8	18	13.1
Perceived stress symptoms								
None	9897	50.7	7144	32.0	156	43.1	35	25.5
1/week	4181	21.4	5129	23.0	77	21.3	22	16.1
2–3/week	3937	20.2	6442	28.9	77	21.3	47	34.3
4–8/week	1494	7.7	3609	16.1	52	14.3	33	24.1
Self-rated health								
Very good	7465	38.3	6233	27.9	117	32.3	27	19.7
Average or good	11,637	59.6	15,568	69.7	229	63.3	99	72.3
Poor	328	1.7	458	2.1	13	3.6	11	8.0
No data	79	0.4	65	0.3	3	0.8	0	0.0
Health-promoting behaviour								
Physical activity								
Very active efficient exerciser	5114	26.2	3930	17.6	84	23.2	13	9.5
Active efficient exerciser	6017	30.9	6623	29.7	105	29.0	44	32.1
Occasional/low efficient exerciser	4645	23.8	7224	32.3	78	21.5	44	32.1
Does not exercise	3671	18.8	4503	20.2	93	25.7	36	26.3
No data	62	0.3	44	0.2	2	0.6	0	0.0
Regular tooth brushing								
Several times/day	3982	20.4	10,831	48.5	53	14.6	69	50.4
About once/day	9737	49.9	9689	43.4	161	44.5	54	39.4
About 1–5 times/week or less	5689	29.2	1754	7.9	145	40.1	14	10.2
No data	101	0.5	50	0.2	3	0.8	0	0.0
Social support								
Nuclear family (with both parents)								
Yes	15,366	78.8	17,040	76.3	250	69.1	96	70.1
No	4022	20.6	5173	23.2	106	29.3	40	19.2
No data	121	0.6	111	0.5	6	1.6	1	0.7
Talking about issues to father								
Easy	10,421	53.4	8157	36.6	156	43.1	38	27.7
Difficult	6010	30.8	8470	37.9	115	31.8	49	35.8
Very difficult/No father	2571	13.2	5314	23.8	80	22.1	47	34.3
No data	507	2.6	383	1.7	11	3.0	3	2.2
Talking about issues to mother								
Easy	13,705	70.3	16,235	72.7	226	62.4	87	63.5
Difficult	4429	22.7	4743	21.2	100	27.6	32	23.4
Very difficult/No mother	1037	5.3	1175	5.3	31	8.6	16	11.6
No data	338	1.7	171	0.8	5	1.4	2	1.5
Talking about issues to friends								
Easy	14,764	75.7	20,078	89.9	258	71.3	120	87.6
Difficult	3558	18.2	1772	7.9	69	19.0	14	10.2
Very difficult/No friends	762	3.9	288	1.3	26	7.2	1	0.7
No data	425	2.2	186	0.9	9	2.5	2	1.5
School achievement								
Highest	3217	16.5	5481	24.6	29	8.0	27	19.7
2nd highest	5563	28.5	7590	34.0	82	22.6	37	27.0
2nd lowest	6993	35.8	6482	29.0	148	40.9	34	24.8
Lowest	3400	17.9	2577	11.5	101	27.9	37	27.0
No data	246	1.3	194	0.9	2	0.6	2	1.5

Family SES was based on parents’ education from Statistics Finland categorized into basic, secondary and high. Data was obtained nearest to the year when the adolescent was aged 15 years. If parents belonged to different categories, the highest was selected. If one parent was missing (2%), the available parent’s data was used.

Within each dimension of reserve capacity (survey data), correlations and associations of the variables were calculated. Moderate positive correlations (Spearman’s) and statistically significant associations (Pearson chi-square tests) ensured that they measured the same dimension.

- *Perceived health* included three items: has a chronic disease, injury or disability that restricts daily activities (no/yes); a summary index of weekly perceived stress symptoms (stomachaches, tension or nervousness, irritability or outbursts of anger, trouble falling asleep or waking at night, headache, trembling of hands, feeling tired or weak, feeling dizzy) categorized as having none, 1 symptom/week, 2–3/week and 4–8/week; and, self-rated health categorized as very good, good to average, poor.
- *Health-promoting behaviour* included frequency of tooth brushing (several times a day, once a day, 1–5 times/week or less) and efficiency of physical activity. Efficiency of physical activity was measured by combining information from two variables: frequency of physical activity in leisure time and intensity of exercise (shortness of breath/sweating). This combination used the following categories: does not exercise, exercises with low/occasional efficiency, active efficient exerciser, very active efficient exerciser.
- *Social support* was measured by four items: nuclear family (living with both parents or not); ease of talking about troubling issues to father, to mother and to friends (easy, difficult, very difficult). Those who did not have a father (5%), mother (1%) or friends (0.5%) were set to “very difficult.”

For school achievement, adolescents were categorized as having: highest, 2nd highest, 2nd lowest or lowest academic achievement. The respondents were asked to assess whether their end-of-term school report was much better, slightly better, average, slightly poorer or much poorer than the class average. For 12–14-year-olds (all in comprehensive schools), the last two were combined. For 16–18-year-olds, the first two were further combined and school status (high school/vocational school/not attending school) was additionally used. Respective categories included: high school, better than class average; vocational school, better or high school, average; vocational school, poor to average or high school, poor; and, not at school.

### Statistical analysis

Cox proportional hazards models, stratified by sex, were fitted to determine the relationship of predictor variables with mortality and calculate hazard ratios (HR) with 95% confidence intervals (CIs). Adherence to the proportional hazards assumption was checked using log-log survival curves and a formal significance test based on the unscaled and scaled Schoenfeld residuals [30]. First, a crude model, which considered family SES, each reserve capacity dimension and school achievement, was fitted to analyse each predictor’s unadjusted effect on mortality risk (Model 1). Then, to study whether the reserve capacity variables modified the relationship between SES and mortality, all statistically significant (*p* < 0.05) reserve capacity variables together with SES were included in a backward selection procedure until none could be deleted from the model (Model 2). Finally, school achievement was added (Model 3). An interaction term between family SES and school achievement was also tested. Model fit was assessed using likelihood ratio tests and Akaike Information Criteria (AIC) [31]. Post-estimation tests were done (checking of residuals and other plots) to ensure that the final model had the best fit. Respondents with missing data (5%) in one or more main variables studied were dropped from analysis. All analyses were performed using STATA version 12.1.

## Results

### Characteristics of the study population

Table 1 lists the detailed characteristics of the total population according to family SES, dimensions of reserve capacity, school achievement and outcome status. Less than one-fourth of boys and girls had low family SES. Generally, most adolescents had positive reserve capacity characteristics but boys and girls differed in terms of perceived stress symptoms and tooth brushing frequency, which were more common among the girls, and ease of talking about issues to father, which was more common among the boys. High achievement in school was also more common among the girls compared to boys.

Among boys, with 358,787 person-years of follow-up time (mean 18.4 years), mortality rate was 10.1 per 10,000 population. In contrast, mortality rate among the girls with 411,373 person-years of follow-up time (mean 18.4 years), was lower (*p* < 0.001) at 3.3 per 10,000 population.

### Predictors of mortality in adolescent boys

Table 2 shows that family SES was significantly and inversely associated with risk of mortality in boys (Model 1), even when the effects of reserve capacity (Model 2) and school achievement (Model 3) were taken into account. Adjusted estimates showed that all reserve capacity dimensions were significant predictors of mortality. Increased risks of death were particularly observed among those with a chronic disease (HR 1.6, 95% 1.2–2.1) and many (4–8) stress symptoms (HR 1.7, 95% 1.2–2.3), those not brushing their teeth daily (HR 1.5, 95% 1.0–2.0), those without a nuclear family (HR 1.4, 95% 1.0–2.7) and those who cannot talk to father easily (HR 1.6, 95% CI 1.2–2.1). All categories below the highest school achievement strongly predicted the risk of mortality, even in the presence of other predictors. The interaction term between family SES and school achievement was not statistically significant.

**Table 2:** Cox proportional hazards models for the effect of socioeconomic status, reserve capacity variables and school achievement on mortality in Finland, Boys, Hazard ratios with 95% confidence interval (CI) estimates

Predictor variables in adolescence	Model 1^a^	Model 2^b^	Model 3^c^
Family SES (parents’ education)			
Higher	1.0	1.0	1.0
Secondary	1.6 (1.1–2.4)*	1.5 (1.0–2.1)*	1.3 (0.9–1.9)
Basic or lower	2.2 (1.5–3.3)**	1.9 (1.3–2.9)**	1.6 (1.1–2.4)*
Perceived health			
Chronic disease			
No	1.0	1.0	1.0
Yes	1.5 (1.1–2.1)**	1.6 (1.2–2.1)*	1.6 (1.2–2.1)**
Perceived stress symptoms			
None	1.0	1.0	1.0
1/week	1.2 (0.9–1.5)	1.1 (0.8–1.5)	1.1 (0.8–1.5)
2–3/week	1.2 (0.9–1.6)	1.1 (0.8–1.5)	1.1 (0.8–1.5)
4–8/week	1.8 (1.3–2.6)**	1.7 (1.2–2.4)*	1.7 (1.2–2.3)**
Self-rated health			
Very good	1.0		
Average or good	1.2 (0.9–1.5)	n.s.	n.s.
Poor	1.8 (0.9–3.3)		
Health-promoting behaviour			
Physical activity			
Very active efficient exerciser	1.0		
Active efficient exerciser	1.0 (0.7–1.3)	n.s.	n.s.
Occasional/low efficient exerciser	1.0 (0.7–1.3)		
Does not exercise	1.3 (1.0–1.8)		
Regular tooth brushing			
Several times/day	1.0	1.0	1.0
Once/day	1.2 (0.9–1.7)	1.2 (0.9–1.7)	1.1 (0.8–1.6)
1–5 times/week or less	1.9 (1.3–2.6)**	1.7 (1.2–2.3)*	1.5 (1.0–2.0)*
Social support			
Nuclear family (with both parents)			
Yes	1.0	1.0	1.0
No	1.5 (1.2–1.9)*	1.4 (1.1–1.8)*	1.4 (1.0–1.7)*
Talking about issues to father			
Easy	1.0	1.0	1.0
Difficult	1.2 (0.9–1.6)	1.2 (1.0–1.6)	1.2 (1.0–1.6)
Very difficult/No father	1.6 (1.1–2.2)**	1.6 (1.2–2.1)*	1.6 (1.2–2.1)**
Talking about issues to mother			
Easy	1.0		
Difficult	1.2 (0.9–1.6)	n.s.	n.s.
Very difficult/No mother	1.2 (0.7–1.8)		
Talking about issues to friends			
Easy	1.0		
Difficult	1.0 (0.8–1.3)	n.s.	n.s
Very difficult/No friends	1.4 (0.9–2.1)		
School achievement			
Highest	1.0		1.0
2nd highest	1.7 (1.1–2.6)*		1.6 (1.0–2.4)*
2nd lowest	2.4 (1.6–3.6)**	–	2.0 (1.3–3.1)**
Lowest	3.1 (2.0–4.7)**		2.3 (1.4–3.5)**

*p < 0.05; **p < 0.001; n.s. not significant

^a^Model 1. All predictor variables ^b^Model 2. All significant reserve capacity variables from Model 1 and family SES. ^c^Model 3. Model 2 variables and school achievement

Accounting for reserve capacity significantly reduced the effect of low SES on the risk of death, more so when school achievement was controlled for. Among boys whose parents had secondary education, HR estimates decreased by almost 19%, from 1.6 (95% CI 1.1–2.4) to 1.3 (95% CI 0.9–1.9). Total reduction in HR estimates was greater (27%) among those whose parents had basic/lower education, from 2.2 (95% CI 1.5–3.3) to 1.6 (95% CI 1.1–2.4). Interestingly, HR estimates for reserve capacity did not change markedly even with adjustment for the effect of school achievement.

### Predictors of mortality in adolescent girls

There were fewer predictor variables significantly related to risk of mortality in girls (Table 3). Family SES was not associated with girls’ risk of death. Accounting for the effects of family SES and school achievement (Model 3), increased mortality risks were observed among girls with poor perceived health indicated by poor self-rated health (HR 4.5, 95% CI 2.2–9.4) and lack of social support due to difficulty talking with one’s father (HR 1.7, 95% 1.1–2.6). Only the lowest category of school achievement significantly increased their risk of death (HR 2.4, 95% CI 1.4–4.1). As observed in boys, the interaction term between family SES and school achievement was also not statistically significant.

**Table 3:** Cox proportional hazards models for the effect of socioeconomic status, reserve capacity variables and school achievement on mortality in Finland, Girls, Hazard ratios with 95% confidence interval (CI) estimates

Predictor variables in adolescence	Model 1^a^	Model 2^b^	Model 3^c^
Family SES (parents’ education)			
Higher	1.0	1.0	1.0
Secondary	1.0 (0.6–1.8)	1.0 (0.6–1.7)	0.9 (0.5–1.5)
Basic or lower	1.4 (0.8–2.4)	1.2 (0.7–2.1)	1.0 (0.6–1.8)
Perceived health			
Chronic disease			
No	1.0	n.s.	n.s.
Yes	1.2 (0.7–2.0)		
Perceived stress symptoms			
None	1.0		
1/week	0.8 (0.5–1.4)	n.s.	n.s.
2–3/week	1.3 (0.8–2.1)		
4–8/week	1.5 (0.9–2.5)		
Self-rated health			
Very good	1.0	1.0	1.0
Average or good	1.4 (0.9–2.2)	1.4 (0.9–2.2)	1.4 (0.9–2.1)
Poor	4.5 (2.1–9.6)**	5.2 (2.5–10.6)**	4.5 (2.2–9.4)**
Health-promoting behaviour			
Physical activity			
Very active efficient exerciser	1.0		
Active efficient exerciser	1.9 (1.0–3.5)*	n.s.	n.s.
Occasional or low efficient exerciser	1.7 (0.9–3.2)		
Does not exercise	2.0 (1.0–3.8)*		
Regular tooth brushing			
Several times/day	1.0		
Once/day	0.9 (0.6–1.3)	n.s.	n.s.
1–5 times/week or less	1.3 (0.7–2.3)		
Social support			
Nuclear family (with both parents)			
Yes	1.0	n.s	n.s.
No	1.2 (0.8–1.7)		
Talking about issues to father			
Easy	1.0	1.0	1.0
Difficult	1.3 (0.8–2.0)	1.2 (0.8–1.9)	1.3 (0.8–1.9)
Very difficult/No father	1.7 (1.0–2.8)*	1.8 (1.1–2.7)*	1.7 (1.1–2.6)*
Talking about issues to mother			
Easy	1.0		
Difficult	1.0 (0.6–1.6)	n.s	n.s.
Very difficult/No mother	1.9 (1.1–3.5)*		
Talking about issues to friends			
Easy	1.0		
Difficult	1.1 (0.6–1.9)	n.s	n.s.
Very difficult/No friends	0.4 (0.6–3.1)		
School achievement			
Highest	1.0		1.0
2nd highest	1.0 (0.6–1.6)		1.0 (0.6–1.6)
2nd lowest	1.0 (0.6–1.7)	–	1.0 (0.6–1.6)
Lowest	2.8 (1.7–4.7)**		2.4 (1.4–4.1)**

*p < 0.05; **p < 0.001; n.s. not significant

^a^Model 1. All predictor variables ^b^Model 2. All significant reserve capacity variables from Model 1 and family SES. ^c^Model 3. Model 2 variables and school achievement

In the crude model (Model 1), the lowest SES category, although not statistically significant, showed an inverse relationship with mortality (HR 1.4, 95% 0.8–2.4). However, this effect was diluted and HR estimates became null when reserve capacity and school achievement were taken into account. Similar to results seen in boys, HR estimates for reserve capacity did not change markedly even when school achievement was added into the model.

## Discussion

### Summary and interpretation of results

Our study found that family SES in adolescence significantly predicted risk of death only in boys. Among reserve capacity dimensions, poor perceived health (presence of chronic disease and weekly stress symptoms in boys; poor self-rated health in girls) as well as reduced social support (difficulty in talking to father in both groups; not living in a nuclear family in boys) generally increased the mortality risk of adolescents. Poor health-promoting behaviour (poor oral hygiene) increased the risk only in boys. Adolescents with low school achievement had 1.6–2.3 times higher risk of dying compared to the highest achievers. Reserve capacity and school achievement independently mitigated the effects of low SES on mortality risk among boys.

Family SES was related with boys’ mortality risk in adolescence and early adulthood in our study. In Finland, previous research also revealed that health inequalities in adolescence and early adulthood persisted in boys from low SES environments possibly due to risky living standards and lifestyle-related factors [32, 33]. Likewise, studies on adult SES measures and outcomes presented stronger effects of SES on mortality for men relative to women because of underlying gender roles and other social characteristics [6, 10]. Typically, though, socioeconomic differentials in morbidity and mortality were recognised as less salient in the adolescent population compared to adults due to a certain level of “equalisation” of risk exposures [32, 34].

Our findings showed that all reserve capacity dimensions significantly predicted mortality risk in boys. Among girls, similar results were observed, except for health-promoting behaviour. A particular study which found difference in psychosocial resources between teenage boys and girls used a different dimension from those analysed in our study [28]. Thus, we cannot conclusively say that there are gender differentials in reserve capacity. Moreover, most epidemiological studies which dealt with reserve capacity’s role in SES-health inequalities controlled for the effect of sex and combined results for both groups [17, 34, 35].

Since poor health perceptions are usually influenced by the presence of co-morbid conditions and symptoms [36], we included these along with self-rated health in the perceived health dimension. Studies have shown that perceived health was strongly and independently associated with mortality, even after controlling for known risk factors [36, 37], and objective physician ratings [38]. Researchers have explained that this indicator may have a summative property of capturing health aspects relevant to survival which are not measured by other health indicators [37]. In adolescence, health perceptions also reflect one’s overall sense of psychosocial functioning aside from physical health [39]. Based on our results, changing self-perceptions of health and alleviating stress symptoms might improve both psychosocial and physical functioning in adolescence.

In our study, physical activity was not associated with the risk of death. Perhaps, this was because among those who died and regardless of their SES, both boys and girls were physically active in their adolescent years. Such health-promoting behaviour is usually adopted early in life [20] and further reinforced by school environments [40]. However, lack of health-promoting behaviour in terms of poor tooth brushing habits, was associated with boys’ mortality risk. The girls in our study generally had good dental behaviour, hence, there was little variation in the distribution of exposure, unlike in boys. Tooth brushing behaviour, also formed during childhood, probably reflected family conditions, such as how well parents provide care and monitor their children’s health behaviour, to some extent [22].

Research on the effect of social support on mortality was extensive. A meta-analytic review showed that overall effect size of being in social relationships provided up to a 50% increase in odds of survival [24]. In our study, important aspects of social support were related to family structure and communication with father. Researchers have recognized that a “risky” family environment early in life predisposed children to various emotional and physical disorders [9, 17, 34]. In a study among Hungarian adolescents, a non-intact family structure was a significant determinant of risky health behaviours such as use of cigarettes, alcohol and marijuana [34]. Our results showed that poor communication with one’s father increased the mortality risk of adolescents. However, the mechanisms by which communication with one’s father influences health during adolescence is beyond the scope of our study. Nonetheless, our results, comparable to earlier findings [41], underscore the importance of paternal relationship as a form of social support. This is congruent with evidence that showed children had less emotional and behavioral problems with father’s involvement during childhood and adolescence [42].

School achievement also significantly predicted the risk of death in both genders in our study. Previous studies showed that school achievement in adolescence empowered a person to make healthy choices and adopt healthy habits [25, 26]. It also ensured completion of high school education, often leading to a college degree, greatly improving one’s future SES [8, 26]. In our study, increasing mortality risk in boys was estimated with each category below the highest achievement. In girls, only the lowest category was significantly related to risk of death. The lack of interaction between family SES and school achievement implies that both education-related variables exhibit a similar and expected gradient with mortality.

As shown in literature [11, 17, 19], our results demonstrated that reserve capacity reduced the effect of low SES on mortality risk among boys. Interestingly, the addition of school achievement into the model further weakened the effect of low SES on boys’ risk of death. Yet, it did not modify the risk estimates obtained from the reserve capacity dimensions, suggesting that these factors are important predictors which independently affect mortality risks in adolescents. The results of our study lend further support for the life-course approach to the SES-health relationship.

### Strengths and weaknesses

Most studies have utilized either childhood or adult markers of SES. Adolescent indicators are seldom used, even though adolescence is a critical period in developing sound psychological and behavioural patterns, which are carried forward into adulthood [28]. Our prospective study addressed this research gap using large, nationwide samples with a long follow-up period and reliable register-based data. Our study added support to the importance of the life-course approach in epidemiologic research on SES-health inequalities.

Studies which dealt with a reserve capacity framework among adolescents were limited. The opportunity to combine survey data with register-based data on death made it possible to build a longitudinal dataset and study potential psychosocial factors mediating the SES-health gradient. Since the survey data was collected in the 1980s and 1990s, it was not designed to measure dimensions of reserve capacity. Due to this, we needed to use proxy measures for each reserve capacity dimension. The selection of variables was based on a cluster of single-item indicators which correlated with each other. However, proxy measures may give unreliable results and further research is needed to validate these.

Despite issues in measurements, we tried to analyse a wide range of reserve capacity dimensions. This follows the methodological framework of the proponents of reserve capacity who emphasised that it is “a bank of resilient resources that contributes to the SES and health relationship” [9, 17–19] instead of a single psychosocial factor or dimension. Moreover, we presented results disaggregated by sex, providing evidence to the interconnections of SES, gender and health inequalities.

## Conclusions

We found that reserve capacity, measuring psychosocial resources plus health-promoting behaviour, and good school achievement in adolescence reduce the risk of mortality in adolescence and early adulthood. In boys, these also mitigate the negative effect of low SES on mortality. These findings underscore the role of reserve capacity and school achievement during adolescence as likely causal or mediating mechanisms in SES-health inequalities.

## Abbreviations

AHLS: Adolescent Health and Lifestyle Surveys
AIC: Akaike Information Criteria
CI: confidence interval
HR: hazard ratio
OR: odds ratio
SES: socioeconomic status
